# Study of Psychomotor Agitation Constraint Method: A Systematic Review

**DOI:** 10.62641/aep.v53i3.1997

**Published:** 2025-05-05

**Authors:** Shengnan Zhu, Jing Liu, Aili Cao, Fan Jiang, Rui Wang

**Affiliations:** ^1^Department of General Practice, Minhang Hospital, Fudan University, 201100 Shanghai, China; ^2^Department of Nursing, Minhang Hospital, Fudan University, 201100 Shanghai, China; ^3^Department of Emergency Intensive Care Unit, Minhang Hospital, Fudan University, 201100 Shanghai, China

**Keywords:** psychomotor agitation, constraint method, physical restraint, healthcare resources, clinical importance

## Abstract

**Background:**

Psychomotor agitation is a common psychiatric disorder that often requires physical restraint, consuming significant healthcare resources. Assessing the clinical importance of the correct method of physical restraint for patients with psychomotor agitation presents a challenge for physicians and researchers. This review aims to assess the use of physical restraints in Intensive Care Units (ICUs) and other departments, identifying potential factors influencing their use.

**Methods:**

Two independent researchers conducted a computerized search of PubMed, Embase, Web of Science, and Cochrane databases for literature related to methods of psychomotor agitation restraint. The review focused on the methods of inhibiting psychomotor agitation in the ICU.

**Results:**

A total of seven papers met the inclusion criteria for this systematic review. The restraint rates among patients ranged from 8.7% to 59.07%. Factors influencing patient restraint included gender, marital status, mental and behavioral disorders, emergency referrals, and the use of mechanical ventilation.

**Conclusions:**

Restraint is frequently used among patients, particularly among the elderly, males, and those with disorders of consciousness or social relationship issues. This review identifies several factors influencing restraint rates in patients with psychomotor agitation, highlighting the need for further research to develop targeted interventions aimed at reducing the necessity for physical restraints.

## Introduction

Psychomotor agitation refers to the significant increase in behavioral movements and verbal activities. Since behavior is affected by thinking and emotional activities, such patients also have abnormalities in thinking and emotional aspects [1]. Psychomotor agitation is characterized by increased psychomotor activity, agitation, and irritability. Patients in a state of agitation exhibit heightened responsiveness to internal and external stimuli, which is often accompanied by mental tension or changes in cognitive function. This condition is associated with increased risks of self-harm, injury, and poor treatment response [2].

Psychomotor agitation becomes serious over time, and early recognition of psychomotor agitation is crucial for the treatment of patients, the safety of medical staff, and the improvement of patient prognosis [3]. Patients with psychomotor agitation begin to become excited, eye contact, increase responsiveness and alertness to external stimuli, and irritable. Patients with psychomotor agitation further develop verbal growling, violent reactions, uncooperative, or paranoid behavior [4]. Therefore, the use of a constraint method is essential to treat the patients.

The use of restraint methods is a fairly common practice in patients with psychomotor agitation. Restraint methods for inpatients with psychomotor agitation are mainly used to prevent patients from having unexpected events or to control patients who do not cooperate with treatment. Restraint methods for inpatients with psychomotor agitation are used as important nursing measures to prevent patients from falling, removing tubes, self-injury, or injuring others [4]. In Canada, Australia, the United States, and other countries, the restraint rate is relatively high. A multi-center study conducted in 39 hospitals in the United States found that 44% of hospitalized patients had been restrained [5], and the restraint method is used as a routine measure [6]. In China, Zhu *et al*. [7] found that the patient restraint rate was as high as 47.05%. Zhu *et al*. [7] studied 356 patients and found that the constraint rate was 39.04%. According to the survey of patients conducted by Xu and Zheng [8], 131 patients had used restraints, and the restraint rate was 35.1%. The above literature shows that the usage rate of constraint methods varies among countries, but the overall usage rate of constraint methods is relatively high.

However, our review of relevant literature reports also found that patients with psychomotor agitation had different degrees of complications when they chose different restraint methods. The complications arising from the restraint methods of hospitalized patients with psychomotor agitation mainly include skin complications at the restraint site, delirium, post-traumatic stress disorder, and resulting in prolonged stay in even death in severe cases [9, 10, 11, 12, 13, 14]. It indicates that there are some problems in the selection of restraint methods for psychomotor agitation in hospitalized patients. More and more clinical scholars and institutions in many countries began to pay close attention to and re-examine the rationality and correctness of the use of restraint methods for inpatients with psychomotor agitation, and we also actively carried out relevant studies on restraint methods. We conducted a systematic literature review and summary of the research results of restraint methods for psychomotor agitation in relevant inpatients, solved the inconsistency of the results of various studies, and reached a reasonable conclusion. Exploring the restraint methods of psychomotor agitation can standardize the use of restraint methods of psychomotor agitation.

The study aims to collect, integrate, and analyze existing literature to explore the impact of different constraint methods on the efficacy and safety of patients with psychomotor agitation. By systematically synthesizing the findings of relevant studies, the study aims to provide a comprehensive evaluation and conclusion regarding constraint methods for psychomotor agitation, to guide clinical practice.

## Materials and Methods

### Search Strategy

The main and abstract checklist of PRISMA were completed (**Supplementary Table 1**). Entries in PubMed, Embase, Scopus, Web of Science, and Cochrane databases were searched by computer between January 2000 and August 2023. To ensure the recall and accuracy of the references, fuzzy search was carried out to collect the references that met the inclusion criteria. Search the terms are: restraint AND delirium OR agitated OR psychomotor agitation.

### Inclusion and Exclusion Criteria of Literature

Inclusion criteria: a cross-sectional study, cohort study, or case-control study; Patients with psychomotor agitation; ≥18 years old; Research content including incidence of constraints, factors affecting constraints, methods of constraints, effects of constraints, etc. Languages were English and Chinese. Exclusion criteria: there is too little important information in the literature report, or the full text is not obtained through various channels, only the abstract literature.

Two researchers with evidence-based learning and training conducted a comprehensive and systematic search of each English database and related guide websites. Disagreements are resolved through discussion, concerning the opinion of an independent arbiter as needed. In addition, the “snowball” search method is used to track down the references after inclusion. The document management software NoteExpress (version 3.8.0.9520, Beijing Aegean Technology Co., LTD., Beijing, China) was used to delete and sort the repeated publications. Use PRISMA flowcharts to document search strategies and research selection processes.

### Quality Assessment

The Critical Appraisal Skills Program (CASP) checklist is used to assess observational studies, primarily cohort studies and case-control studies. The checklist for evaluating cohort studies consists of 12 questions, with the first two being screening questions and the remaining ten being detailed questions. The checklist for evaluating case-control studies consists of 11 questions, with the first two being screening questions and the remaining nine being detailed questions. The 12 questions can be divided into three parts for evaluating the findings, their reliability, and their applicability. Questions 1 to 7 and 10 to 12 are answered with “yes”, “no”, or “don’t know”. Articles 8 and 9 require evaluation based on the cohort study method, causal inference rule, and correlation strength test.

### Data Extraction and Synthesis of Evidence

The extracted data includes the following research information: authors, year of publication, and country. The characteristics of the subjects include gender composition, average age, and the unit they belong to (e.g., intensive care unit, psychiatric department, etc.). The data also includes the restraint rate among the patients. The patients’ diseases encompass schizophrenia, mania, delirium, cerebral hemorrhage, among others. The various restraint methods employed are bed railings, belt fixation, electronic restraint devices, and physical restraints, among others.

## Results

### Literature Screening Results and Related Information on Restraint Methods

**Fig. 1.**
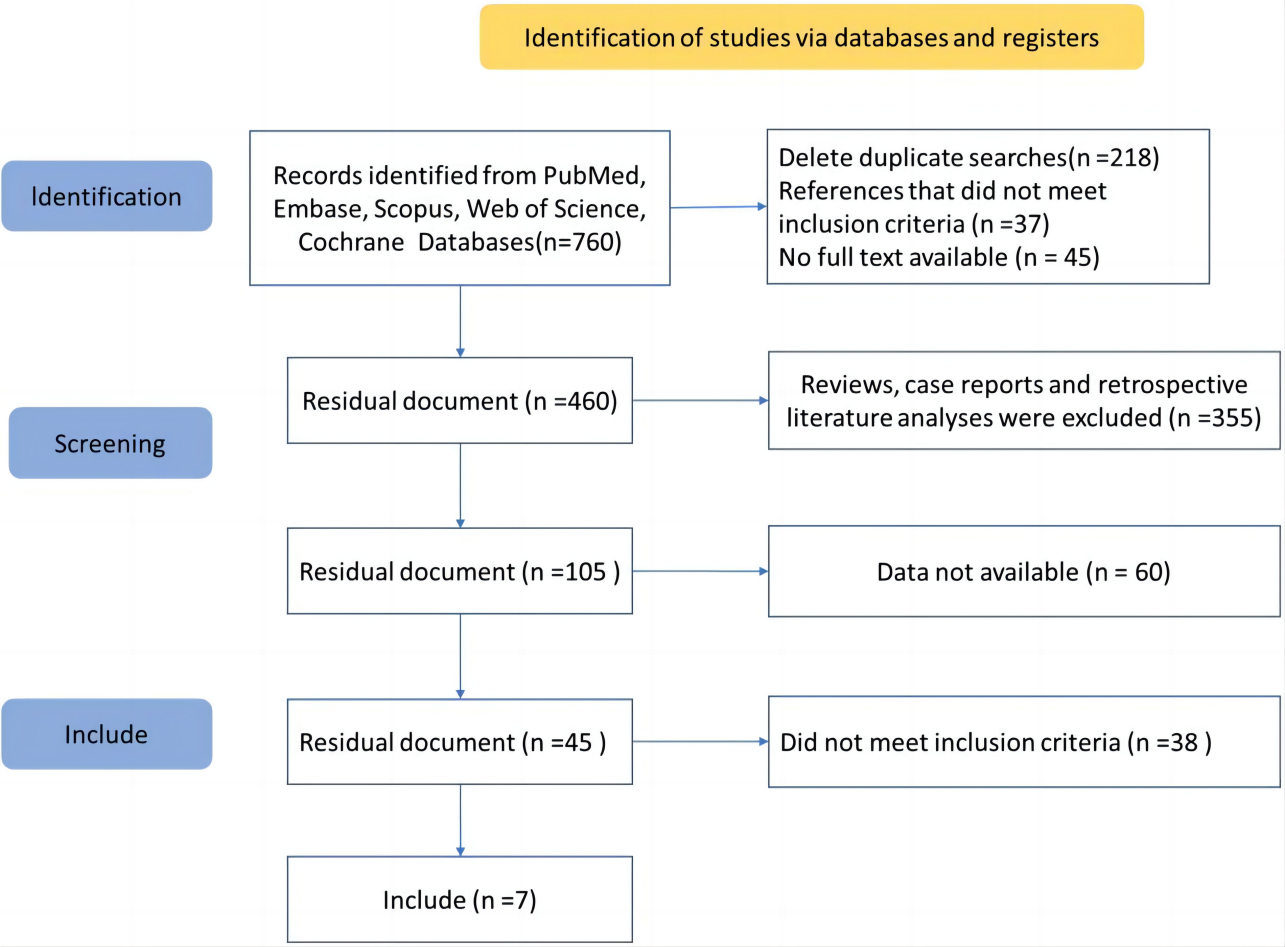
**Flow diagram of study selection process**.

The research screening process, as depicted in Fig. 1, involved the exclusion of 218 duplicate literatures. Following this, a selection was made based on the title and abstract of the remaining literatures. Two independent reviewers screened the articles, leading to the exclusion of 37 articles that did not meet the inclusion criteria and 45 articles without full texts. Additionally, 355 articles were excluded due to their irrelevance to the study’s focus. From the 105 re-listed papers, 60 were further excluded for not providing complete information, and 38 were excluded for not meeting the inclusion criteria. Ultimately, seven papers were included in the systematic evaluation, which are referenced as [9, 15, 16, 17, 18, 19, 20]. The included studies spanned various countries, including Egypt, China, Brazil, Switzerland, and Austria, and covered different settings such as Intensive Care Units (ICU), home care, and old-age psychiatry inpatient units. The study designs ranged from cross-sectional to nested case-control and retrospective cohort studies. The number of patients across these studies varied significantly, from 162 in a home care setting to 29,477 in a cross-sectional study across Switzerland and Austria. The restraint rates reported in these studies also showed a wide range. The influence factors associated with the use of physical restraints were diverse (Table 1, Ref. [9, 15, 16, 17, 18, 19, 20]).

**Table 1.** **Basic characteristics of the included literature**.

Author	Time	Country	Study type	Patients	Gender (male/female)	Unit	Restraint rate	Influence factor
Kandeel and Attia [9]	2013	Egypt	A cross-sectional study	275	154/121	ICU	-	Type of disease, patients’ behavior, individuals responsible for the decision of applying physical restraints.
Pan *et al*. [15]	2018	China	A nested case-control study	356	254/102	ICU	-	-
Capeletto *et al*. [20]	2021	Brazil	A cross-sectional study	162	64/98	Home care	13.0%	Caregiver routine on alternate days, walks, bed restriction, permanent bladder catheterization.
Thomann *et al*. [16]	2021	Switzerland Austria	A cross-sectional study	29,477	14,973/14,504	-	8.7%	Country, age (year), female, type of disease.
Cui *et al*. [17]	2023	China	A retrospective cohort study	3776	2455/1321	ICU	48.8%	Male, surgical ICU, pain, light sedation, muscle strength, ICU length of stay, tracheal tubes, abdominal drainage tubes.
Chieze *et al*. [18]	2021	Switzerland	A cross-sectional study	494	207/287	Old-age psychiatry inpatient units	16.4%	Male, age (year), marital status, total number of psychiatric hospitalizations, referrals from the emergency department, involuntary admission, admission Health of the Nation Outcome Scales (HoNOS) item 1 (overactive, aggressive, disruptive or agitated behavior), cognitive disorder.
Zhang *et al*. [19]	2021	China	A cross-sectional study	386	267/119	ICU	59.07%	Male, mechanical ventilation, retained catheters or tubes with level II, irritability.

ICU, Intensive Care Unit.

### Quality Evaluation of Included Literature

The methodological evaluation results of 7 papers are shown in Table 2 (Ref. [9, 15, 16, 17, 18, 19, 20]). All of the population data resources and inclusion and exclusion criteria were explicit. Confounding factors were reasonably controlled, but there were fewer studies explicitly reported the study quality control and handling of missing data. We assessed seven papers using various criteria, and the findings revealed that two papers fulfilled all seven question criteria, three papers met six criteria, one paper met five criteria, and one paper met four criteria. Overall, the quality of the papers was high.

**Table 2.** **The result of literature quality evolution**.

Literature	1	2	3	4	5	6	7	8	9	10	11
Kandeel and Attia [9]	Y	Y	Y	NA	NA	N	N	Y	Y	N	N
Pan *et al*. [15]	Y	Y	Y	NA	NA	N	N	Y	N	N	N
Capeletto *et al*. [20]	Y	Y	Y	NA	NA	N	Y	Y	N	Y	N
Thomann *et al*. [16]	Y	Y	Y	NA	NA	N	Y	Y	N	Y	N
Cui *et al*. [17]	Y	Y	Y	NA	NA	N	Y	Y	N	Y	N
Chieze *et al*. [18]	Y	Y	Y	NA	NA	N	Y	Y	Y	Y	N
Zhang *et al*. [19]	Y	Y	Y	NA	NA	N	Y	Y	Y	Y	N

**Note: **(1) Whether the source of the literature was identified (investigation, review); (2) Whether the inclusion and exclusion criteria was listed; (3) Whether the period of recognizing patients was showed; (4) If were not the population resource, whether the subjective was continue; (5) Whether the other situation of the subjective were covered by the subjective factors from valuator; (6) Any evaluations for guarantee quality were described; (7) The reason of excluded patients were explained; (8) The measure of evaluating or controlling the confounding factor; (9) How to dispose the missing data was explained; (10) Summarizing the response of patients and the integrality of data; (11) Follow-up results. Y means “compliant with standards”, N means “substandard”, NA means “not applicable”.

### Comparison of Patient Restraint Rate

The patient restraint rate of the seven included literature ranged from 8.7% to 59.07% (Table 1, Ref. [9, 15, 16, 17, 18, 19, 20]). The restraint rates reported in these studies showed a wide range, with the highest rate of 59.07% observed in an ICU setting in China. Chieze *et al*. [18] reported that of 494 patients, 81 (16.4%) experienced at least one restraint during hospitalization. Men with cognitive impairment, aggressive behavior, and previous psychiatric hospitalizations had a higher risk of being restrained. Thomann *et al*. [16] reported a total of 29,477 patients enrolled in 140 hospitals, and restraint use was documented in patient files in 8.7% of cases (n = 2577). Hospitals often use restraints in complex care situations, such as when patients are at risk of falling or delirium. In hospitals, restraints are often used in complex care situations. However, their use does not appear to have been adequately documented and evaluated.

### Influencing Factors of Patient Restraint

We proceeded with a descriptive analysis of the influencing factors of patient restraint. For patient demographic data, 3 results showed that elderly male patients had a high rate of restraint [17, 18, 19], and 1 paper found that patients from different countries had various rates of restraint [16]. For the information of diseases, patients with mental and behavioural disorders had a high rate of restraint [16, 18]. In addition, patients transferred from emergency were restraint frequently [18]. For the treatment, patients treated with mechanical ventilation [19], retained catheters or tubes with level II [17, 19, 20] received restraint frequently. As for the social relationship, 1 paper found that the status of separation or divorce was related to the rate of patient restraint [18], another paper suggested that the high rate of nurses/patients and family accompanying helped might reduce the application of restraint [9].

In summary, the influencing factors of patient restraint include elderly male patients, patients from different countries, patients with mental and behavioural disorders, patients transferred from the emergency department, mechanical ventilation, retained catheters or tubes with level II as well as the marital status.

## Discussion

In the seven studies we included, patient restraint rates varied between 8.7% and 59.07%. Chieze *et al*.’s study [18] noted that 16.4% of patients experienced at least one restraint during their hospital stay. Next, Thomann *et al*. [16] showed that of 29,477 patients admitted to the hospital, 8.7% of cases had a record of restraint use in their patient files. The study of Capeletto *et al*. [20] is consistent with our results.

According to a study by Capeletto *et al*. [20], the incidence of mechanical restraint in elderly people in-home care is 13%. The study also noted that arms, legs, and chest were the most common areas where mechanical restraints were used, while bandages, tissues, and sheets were the common restraints. Controlling aggressive behavior in the elderly, preventing falls and protecting the elderly are the main reasons for the use of mechanical restraints. In addition, the study found that 42.9 percent of all older adults who participated in the study were confined to more than 24 hours, while 85.7 percent were confined to one room. From these findings, it can be seen that the use of mechanical restraints is relatively common in-home care for the elderly.

However, there are some problems with the use of mechanical restraints. First, the use of mechanical restraints may limit the freedom and quality of life of older adults. Secondly, decisions using mechanical restraints may not be scientific and rational enough, leading to situations of abuse. Therefore, it is necessary to strengthen the management and supervision of mechanical restraints on home care for the elderly to ensure the rationality and safety of their use. From these findings, it can be seen that patient restraint is a common care measure in hospitals. However, these studies also reveal some problems. First, the use of constraints is not adequately documented and evaluated, making it difficult to assess their effectiveness and safety. Second, there appear to be no clear guidelines for decisions that constrain use, and there may be instances of abuse. Therefore, it is necessary to strengthen the management and supervision of patient restraints to ensure the rationality and safety of their use.

Patient restraint is a common measure of care in hospitals, but its use has not been adequately documented and evaluated. To ensure the safety and rights of patients, it is necessary to strengthen the management and supervision of patient restraint. Further research and the development of guidelines can help regulate the use of constraints and reduce abuse.

We found that male patients in neurology ICU are more likely to be physically restrained. This is consistent with the reported results of Rosso *et al*. [1]. These discrepancies could be related to the nature of the characteristics of patients admitted to each unit. Moreover, patients treated with mechanical ventilation, retained catheters or tubes with level II received restraint frequently. In additional, the result showed that the high rate of nurses/patients reduced the restraint. Previous study has showed that 8% of nurses said that when there is insufficient manpower, more patients will be physically restrained to ensure medical safety [2].

The systematic analysis demonstrated the potential impact of multiple factors on patient restraint use. First, it was found that older male patients showed a trend toward higher rates of restraint use, which is related to their possible health needs, behavioral responses, or other factors [21]. In addition, there were significant differences in patient restraint rates across countries, which may reflect differences in cultural, legal, or healthcare systems. In terms of illness, patients with mental and behavioral disorders show higher rates of restraint use, which may reflect the complexity of their conditions and unpredictable behavior [22]. Patients referred from acute care were also more frequently restrained, which may be related to their acute medical condition or emotional state. Patients undergoing mechanical ventilation or retaining tubes during treatment showed significantly higher rates of restraint use, reflecting the fact that these treatments may increase patients’ motor limitations or safety risks and require additional protective measures. In addition, social relationship factors have been found to be associated with restraint use rates. For example, a high percentage of family members or chaperones may contribute to a reduction in restraint use, which may reflect the fact that patients feel more secure and supported when accompanied by family members [23].

Appropriate medication can effectively calm and stabilize patients experiencing agitation, but its use must be tailored to each patient’s specific needs and under the guidance of medical professionals. Different medications may yield varying effects and potential side effects. Similarly, individualized approaches to restraint are crucial as agitation symptoms can vary widely among patients. Care providers should carefully assess each patient’s physical health, medical background, and family situation to determine the most suitable restraint method. Throughout the process, ensuring the patient’s comfort both physically and psychologically is paramount.

## Conclusions

In summary, different restraint methods have different effects on patients with agitation. When selecting the restraint method, the specific situation of the patient should be considered comprehensively, and the position of the patient during the restraint period should be natural and comfortable. This requires the expertise and experience of doctors and caregivers, as well as the cooperation and understanding of patients and families.

## Availability of Data and Materials

The data that support the findings of this study are available on request from the corresponding author, upon reasonable request.

## Supplementary Material

Supplementary material associated with this article can be found, in the online version, at https://doi.org/10.62641/aep.v53i3.1997.
